# Optimizing the Solvent Selection of the Ultrasound-Assisted Extraction of Sea Buckthorn (*Hippophae rhamnoides* L.) Pomace: Phenolic Profiles and Antioxidant Activity

**DOI:** 10.3390/foods13030482

**Published:** 2024-02-02

**Authors:** Dan Wu, Zhihao Yang, Jiong Li, Huilin Huang, Qile Xia, Xingqian Ye, Donghong Liu

**Affiliations:** 1 National-Local Joint Engineering Laboratory of Intelligent Food Technology and Equipment, Zhejiang Key Laboratory for Agro-Food Processing, Integrated Research Base of Southern Fruit and Vegetable Preservation Technology, Zhejiang International Scientific and Technological Cooperation Base of Health Food Manufacturing and Quality Control, Fuli Institute of Food Science, College of Biosystems Engineering and Food Science, Zhejiang University, Hangzhou 310058, China; 22313090@zju.edu.cn (Z.Y.); 0922121@zju.edu.cn (H.H.); cookxql@163.com (Q.X.); psu@zju.edu.cn (X.Y.); dhliu@zju.edu.cn (D.L.); 2 Hangzhou Institute for Food and Drug Control, Hangzhou 310022, China; jokelee2@126.com; 3 Key Laboratory of Post-Harvest Handling of Fruits, Food Science Institute, Zhejiang Academy of Agricultural Sciences, Hangzhou 310021, China

**Keywords:** sea buckthorn pomace, solvent extraction, antioxidant activity, UPLC-MS/MS

## Abstract

Sea buckthorn pomace (SBP) is a by-product of sea buckthorn processing that is rich in bioactive compounds. In this study, different active ingredients were extracted by using different solvents (water, methanol, ethanol, glycerol, ethyl acetate, and petroleum ether) combined with an ultrasonic assisted method. The correlation between the active ingredients and antioxidant properties of the extract was studied, which provided a research basis for the comprehensive utilization of SBP. This study revealed that the 75% ethanol extract had the highest total phenolic content (TPC) of 42.86 ± 0.73 mg GAE/g, while the 75% glycerol extract had the highest total flavonoid content (TFC) of 25.52 ± 1.35 mg RTE/g. The ethanol extract exhibited the strongest antioxidant activity at the same concentration compared with other solvents. The antioxidant activity of the ethanol, methanol, and glycerol extracts increased in a concentration-dependent manner. Thirteen phenolic compounds were detected in the SBP extracts using UPLC-MS/MS analysis. Notably, the 75% glycerol extract contained the highest concentration of all identified phenolic compounds, with rutin (192.21 ± 8.19 μg/g), epigallocatechin (105.49 ± 0.69 μg/g), and protocatechuic acid (27.9 ± 2.38 μg/g) being the most abundant. Flavonols were found to be the main phenolic substances in SBP. A strong correlation was observed between TPC and the antioxidant activities of SBP extracts. In conclusion, the choice of solvent significantly influences the active compounds and antioxidant activities of SBP extracts. SBP extracts are a valuable source of natural phenolics and antioxidants.

## 1. Introduction

Sea buckthorn (*Hippophae rhamnoides* L.) is a thorny deciduous shrub or small tree of the genus Hippophae, widely distributed in Eurasia in the northern hemisphere [1,2]. China is the largest grower and producer of sea buckthorn, and has the richest sea buckthorn germplasm resources [3]. Sea buckthorn has a long history of use in traditional medicine in Europe and Asia, particularly in Tibet, Mongolia, and India, for treating ulcers, coughs, skin diseases, cardiovascular diseases, etc. [4,5,6]. Sea buckthorn has been extensively used in the production of food, health supplements, feed, cosmetics, and pharmaceuticals due to its biological activity and nutritional value [7,8].

Sea buckthorn berries are a rich source of bioactive substances with physiological and nutritional benefits, including antioxidant, anti-inflammatory, antimicrobial, and immunomodulatory properties [9,10]. They are commonly used as food ingredients in the production of beverages, jams, yoghurts, wines, liqueurs, and other products [3,11]. Sea buckthorn pomace is a by-product of fruit juice extraction and contains a variety of nutrients and bioactive substances, including proteins, unsaturated fatty acids, dietary fiber, phenolic acids, flavonoids, sterols, tocopherols, and carotenoids [12,13,14]. As the sea buckthorn industry continues to expand, the pomace has become a valuable resource for recycling and utilization [15,16,17]. Currently, the majority of SBP is discarded as waste, with only a small portion being used as animal feed. This results in a significant waste of resources. However, there are few studies on the utilization of SBP resources [18,19,20].

Medicinal plants, as an important source of natural antioxidants, have attracted attention for their active components and application value. The research on the extraction of bioactive compounds from natural plants has been growing [21]. To fully utilize SBP, it is crucial to select an efficient extraction method to obtain abundant bioactive substances. Ultrasound-assisted extraction is an efficient, easy-to-obtain and cost-effective method for extracting active compounds from plant materials [22,23]. This method utilizes ultrasound bubbles to rupture cell walls, in addition to the vibrational effect of ultrasound enhancing the release and diffusion of intracellular substances, resulting in increased substance release into the solvent [24,25]. Previous research has shown that the ultrasound-assisted extraction of mushroom by-products can result in a 46% increase in phenolic compounds and a 25% increase in antioxidant activity [26]. In a study comparing the recovery of phenolic compounds from grape skins using mechanical agitation extraction, ultrasound-assisted extraction, and microwave-assisted extraction, ultrasound-assisted extraction was found to have the highest phenolic recovery [27]. Furthermore, research has demonstrated that ultrasound-assisted treatment is highly effective in extracting flavonoids, polysaccharides, and glycosides from plant samples [28,29]. Some reports has indicated that an extraction time of approximately 30 min is optimal for the ultrasound-assisted extraction of phenolics [30,31,32].

The choice of extraction solvent significantly influences extraction efficiency. Water, a prevalent solvent, is utilized for flavonoid recovery from SBP [33]. Methanol has been employed for extracting flavonol glycosides from sea buckthorn berries [34]. However, an aqueous 70% methanol solution demonstrated a superior extraction of total phenols from SBP compared to both water and methanol [35]. Recently, it was reported that sea buckthorn polyphenols obtained from 70% methanol extraction exhibit anti-allergy activity [36]. Ethanol, another commonly used solvent, was applied for the sequential extraction of defatted SBP, and it was found that ethanol extraction gave higher yields but the extracts had lower antioxidant activity than the water extracts [37]. A study using 50% ethanol for SBP extraction revealed an enhanced extraction of phenols and flavonoids through ultrasonication [38]. Guo et al. used ethyl acetate to extract and evaluate bound phenolics in four different species of sea buckthorn [39]. Additionally, the extraction of lipophilic substances from sea buckthorn was reported using a mixture of petroleum ether, methanol and ethyl acetate [40]. Water, methanol, and ethanol are commonly used solvents for the proliferative use of SBP. However, due to the different sea buckthorn species used in various reports, a systematic comparison of extraction solvent efficiencies is not possible. Comparative studies on the extraction of SBP with different solvents are still lacking.

This study utilized various organic solvents (methanol, ethanol, glycerol, ethyl acetate, petroleum ether) for the ultrasound-assisted extraction of SBP, investigating the active components (TPC, TFC) and antioxidant activity [2,2-diphenyl-1picrylhydrazyl (DPPH) and 2,2′-azino-bis(3-ethylbenzothiazoline-6-sulphonic acid) (ABTS) radical scavenging, and ferric-reducing antioxidant power (FRAP)] of the extracts. Concurrently, UPLC-MS/MS analysis was conducted for phenolic compound identification within the extracts. The correlation between the active components and antioxidant activity was also explored. The findings provide fundamental data for extracting bioactive substances from SBP, offering a theoretical foundation for the comprehensive use of SBP in the food, cosmetics, and pharmaceutical industries.

## 2. Materials and Methods

### 2.1. Plant Material

SBP used in the experiment was the by-product of sea buckthorn berries after juicing, which was provided by Xinjiang DaEr Biotechnology Co., Ltd (Aksu, China). The sea buckthorn fruit used was the “ShenQiuHong” variety and was harvested from Xinjiang Province in China in September 2022. After air-drying, the SBP was stored in refrigerator at −18 °C.

### 2.2. Chemicals

Folin-ciocalteau’s reagent, 2,4,6-Tris(2-pyridyl)-s-triazine (TPTZ), 6-Hydroxy-2,5,7,8-tetramethylchromane-2-carboxylic acid (Trolox) and 43 phenolic compounds’ standards (Eriocitrin, Narirutin, Naringin, Hesperidin, Neohesperidin, Rhoifolin, Diosmin, Quercitrin, Eriodictyol, Didymin, Poncirin, Quercetin, Naringenin, Luteolin, Hesperetin, Kaempferol, Apigenin, Diosmetin, Sinensetin, Nobiletin, Tangeretin, Isosinensetin, 5-O-Demethylnobiletin, Luteoloside, Ferulic acid, Caffeic acid, Chlorogenic acid, Gallic acid, Rutin, Neoeriocitrin, Isosakuranetin, Protocatechuic acid, 4-hydroxy-3-methoxybenzoic acid, p-Hydroxybenzoic acid, p-Coumaric acid, Sinapic acid, 3,5,6,7,8,3′,4′-heptamethoxyflavone, 4′,5,6,7-tetramethylflavone, 4′,5,7,8-tetramethylflavone, Natsudaidain, Epigallocatechin, Isorhamnetin, Myricetin) were acquired from Shanghai Yuanye Bio-Technology Co., Ltd. (Shanghai, China). 2,2-diphenyl-1picrylhydrazyl (DPPH), 2,2′-azino-bis-(3-ethylbenzthiazoline-6-sulphonic acid) (ABTS) and gallic acid were obtained from Shanghai Macklin Biochemical Co., Ltd. (Shanghai, China). The rest of the chemicals were all from Sinopharm Chemical Reagent Co., Ltd. (Shanghai, China). All solvents/chemicals used were analytical or HPLC grade.

### 2.3. The Preparation of SBP Extracts

SBP was ground into a powder using a FW80 high-speed grinder (Tianjin Taisite Instrument Co., Ltd., Tianjin, China) and then sifted through a 60-mesh sieve. The concentrations of solvents used for SBP extraction were determined as 25%, 50%, and 75%, based on commonly used concentrations for organic solvent extraction in various reports [35,38,41,42]. Solutions of varying concentrations of methanol, ethanol, glycerol, ethyl acetate, and petroleum ether were prepared separately. Methanol, ethanol, and glycerol solutions were diluted in water, while ethyl acetate and petroleum ether solutions were diluted in ethanol. Water and ethanol served as the controls. Subsequently, these solutions (50 mL) were mixed individually with sea buckthorn pomace powder (1 g). The mixture was extracted for 30 min by a SK1200B ultrasonic extractor (Shanghai Kudos Ultrasonic Instruments Co., Ltd., Shanghai, China), followed by filtration to obtain the extracts of SBP with different solvent extractions. The extracts of SBP were stored in a refrigerator at −18 °C, and subsequent experiments were conducted at room temperature (25 ± 2 °C).

### 2.4. The Determination of Total Phenolic Content (TPC)

The TPC in different extracts was determined using the Folin–Ciocalteu method with a slight modification [43,44]. The diluted extract of SBP (20% of the original extract, 0.4 mL) was taken and water (2.4 mL) and Folin-Ciocalteau reagent (0.4 mL) were added. After a 6 min reaction, Na_2_CO_3_ solution (10. 5%, *w*/*v*, 1.6 mL) was added, followed by incubation in the dark for 60 min. Subsequently, their absorbance was measured at 760 nm using a DR3900 UV–Vis spectrophotometer (Hach Company, Loveland, CO, USA). Gallic acid (0–240 μg/mL) was used to construct a standard calibration curve (y = 221.27x − 6.2502, R^2^ = 0.9975). The TPC was expressed as mg of gallic acid equivalents (GAE) per g of sample dry weight (mg GAE/g).

### 2.5. The Determination of Total Flavonoid Content (TFC)

The determination of TFC in SBP extracts followed the China National Agricultural Standard NY/T 3903-202 [45], with some modifications. Fivefold diluted SBP extracts (1 mL) were mixed with NaNO_2_ solution (5%, *w*/*v*, 0.5 mL) and incubated for 5 min before Al(NO_3_)_3_ solution (10%, *w*/*v*, 0.5 mL) and NaOH solution (10%, *w*/*v*, 2 mL) were added. After incubation for 20 min, the mixture was filtered through a 0.22 μm nylon syringe filter (Zhejiang ALWSCI Technologies Co., Ltd., Hangzhou, China), and the absorbance was measured at 508 nm. A standard curve was constructed using rutin (0–140 μg/mL), and the equation was y = 392.85x + 0.9075, where R^2^ = 0.9991. The TFC was expressed as mg of rutin equivalents (RTE) per g of sample dry weight (mg RTE/g).

### 2.6. DPPH Radical Scavenging Assay

The DPPH radical scavenging activity was determined according to the methods in references [44,46]. The sixfold diluted extract of SBP (0.1 mL) was mixed with the ethanol solution of DPPH (0.1 mM, 3.9 mL). The reaction was conducted at room temperature in the dark for 30 min, and absorbance of the sample and blank absorbance were measured at 517 nm. The percentage of DPPH radical scavenging was calculated. Additionally, a standard curve for DPPH radical scavenging activity was constructed using Trolox (0–200 μg/mL). The equation of standard curve was y = 0.004x + 0.0011, where R^2^ = 0.9975. The DPPH radical scavenging activity was expressed as mg of Trolox equivalents per g of dry weight (mg TE/g).

### 2.7. ABTS Radical Scavenging Assay

The ABTS radical scavenging activity of SBP extracts was measured based on reported methods, with slight changes [44]. In brief, the ABTS solution was prepared by the mix of 200 mg ABTS and 34.4 mg potassium persulfate with 50 mL distilled deionized H_2_O, leaving the mixture in the dark for about 24 h at room temperature before use. The ABTS solution was diluted with ethanol until it reached the absorbance of 0.7 ± 0.02 at 734 nm. The diluted extract of SBP (10% of the original extract, 0.1 mL) was mixed with the diluted ABTS solution (3.9 mL) and was allowed to react in the dark for 10 min. The ABTS radical scavenging activity of the mixture was determined by calculating the decrease in absorbance measured at 734 nm. A standard curve for ABTS radical scavenging activity was constructed using Trolox (0–200 μg/mL), and the equation was y = 1.3968x + 0.0001, where R^2^ = 0.9984. The ABTS radical scavenging activity was expressed as mg of Trolox equivalents per g of dry weight (mg TE/g).

### 2.8. Ferric Reducing Antioxidant Power (FRAP)

FRAP was measured based on a previous study, with some modifications [44]. The working solution was mixed with sodium acetate buffer (pH 3.6), TPTZ solution (10 mM TPTZ in 40 mM HCl) and iron trichloride solution (20 mM) in a ratio of 10:1:1 (*v*/*v*). Diluted extract of SBP (10% of the original extract, 0.1 mL) was mixed with the working solution (2.4 mL) and diluted with water to a total volume of 5 mL. After 30 min incubation in the dark, the absorbance was measured at 593 nm. A standard curve (y = 0.0006x + 0.0047, R^2^ = 0.9987) was constructed using ferrous sulphate (0–600 μg/mL). The result was expressed as mg of ferrous sulphate equivalents per g of dry weight (mg FE/g).

### 2.9. UPLC-MS/MS

Determination of phenolic compounds in SBP extracts was carried out on ACQUITY UPLC ultra-high performance liquid chromatography and Waters Xevo TQ-XS tandem quadrupole mass spectrometer (Waters, MA, USA).

Chromatographic separation: A Waters CORTECS UPLC C18 column (100 × 2.1 mm, 1.6 µm, Waters, MA, USA) was used, and the mobile phase consisted of solvent A (0.1% formic acid aqueous solution, *v*/*v*) and solvent B (acetonitrile). The column temperature was 40 °C, flow rate was 0.3 mL/min, and the injection volume was 1 µL. Gradient profile was 0–7.0 min, 10%~50% B; 7.0–10.0 min, 50%~80% B; 10.0–10.1 min, 80%~100% B; 10.1–12 min, 100% A; 12–12.1 min, 100%~10% B; 12.1~15.0 min, 100% B. 

Mass spectrometry: An electrospray ionization (ESI) source was utilized with both positive and negative ion detection modes (ESI+/ESI−). The ion source temperature was maintained at 150 °C and the capillary voltage was set at 2.0 kV. The desolvation gas flow rate was set at 600 L/h. 

### 2.10. Statistical Analysis

All experimental measurements were performed in triplicate, and the results are presented as mean value ± standard deviation (SD). The results were all adjusted by subtracting the blank solvent values. The analysis employed analysis of variance (ANOVA) with post hoc Tukey test (at 95% confidence level) and Bonferroni correction to determine significant differences among the means. Pearson correlation analysis was utilized to evaluate the strength of linear correlations between dependent variables, providing correlation coefficients (*r*) and significance (*p*). Significance levels were defined as *p* < 0.05 (*) and *p* < 0.01 (**). Orthogonal projection to latent structure-discriminant analysis (OPLS-DA) and cluster analysis (CA) was performed using the Metware Cloud (https://cloud.metware.cn (accessed on 7 January 2024)).

## 3. Results and Discussion

### 3.1. The Determination of Total Phenolic Content (TPC)

**Figure 1:**
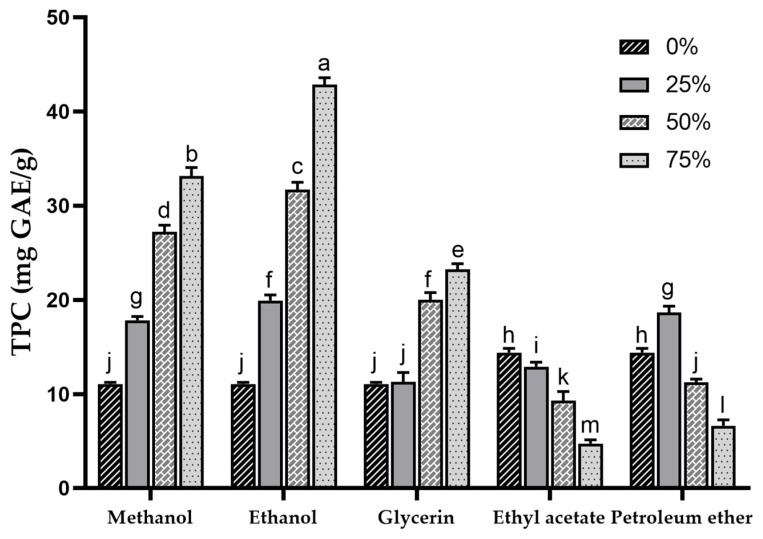
Total phenolic content of sea buckthorn pomace extracts obtained with different solvents. Different lowercase characters represent significant difference at *p* < 0.05 by Tukey’s multiple range test.

Phenolic compounds are important components of plant bioactives and also play an important role in the antioxidant activity of sea buckthorn [1]. In this study, SBP was extracted with different solvents, and the TPC of each extract was determined. The TPC in different concentrations of methanol, ethanol, glycerol, ethyl acetate and petroleum ether extracts are shown in Figure 1. The results indicated extremely significant differences (*p* < 0.001) in TPC among extracts with different types and concentrations of solvents, ranging from 6.63 ± 0.63 mg GAE/g (75% petroleum ether) to 42.86 ± 0.73 mg GAE/g (75% ethanol). The sequence of TPC in the extracts (in terms of mean values) was ethanol > methanol > glycerol > petroleum ether > water > ethyl acetate. This result was substantially higher than the previously reported TPC of sea buckthorn pomace extracted with methanol (2.79 ± 26.6 mg GAE/g) [14] and was comparable to the TPC results of different sea buckthorn varieties investigated in another report (27.6 ± 1.9~38.7 ± 1.3 mg GAE/g) [39]. Kuhkheil et al. reported the TPC of 20.78~34.60 mg/g for wild sea buckthorn populations in Iran [47]. Compared to the reported phenolic content of apple pomace (5.6 mg GAE/g) [48], SBP exhibits superior potential in phenolic extraction.

At the same concentration, higher a TPC was observed in methanol and ethanol extracts, and within the 0–75% concentration range, a concentration-dependent increase in TPC for both methanol and ethanol extracts was noted. This suggests that compared to water, methanol and ethanol exhibit higher efficiency in extracting phenolic compounds from SBP. Other studies have also shown that the highest extraction rate of phenolics was achieved when the concentration of organic solvent was around 75% [49,50,51]. Water with high polarity extracts fewer phenolic compounds with low polarity [52], resulting in a lower TPC in the water extract of SBP.

The TPC in the 25% glycerol extract (11.28 ± 0.99 mg GAE/g) was similar to that in water extract (11.05 ± 0.18 mg GAE/g), whereas with increasing glycerol concentrations, the TPC in the extract gradually increased (23.23 ± 0.60 mg GAE/g). As the concentration of ethyl acetate and petroleum ether increased, the TPC showed a decreasing trend, which may be due to the fact that ethyl acetate and petroleum ether extracted fewer phenolics, and the TPC may be mainly affected by the ethanol acting as a diluent in the extraction solvent.

The extraction efficiency of TPC is influenced by the properties of the extracting solvents, including their ability to act as dipoles, proton acceptors, or proton donors interacting with solutes [53]. The solubility of phenolic compounds is determined by structural properties such as the position of the hydroxyl group and the molecular size and length of the hydrocarbon chain [54]. Lower TPC in water, ethyl acetate, and petroleum ether extracts may indicate that excessively high or low polarity is unfavorable for the extraction of phenolic compounds in SBP. Previous research has shown that the addition of a certain proportion of water to organic solvents can aid in the desorption and dissolution of phenolic compounds, resulting in higher yields [55]. This finding may explain the poor TPC observed in the ethyl acetate and petroleum ether extracts.

### 3.2. The Determination of Total Flavonoid Content (TFC)

**Figure 2:**
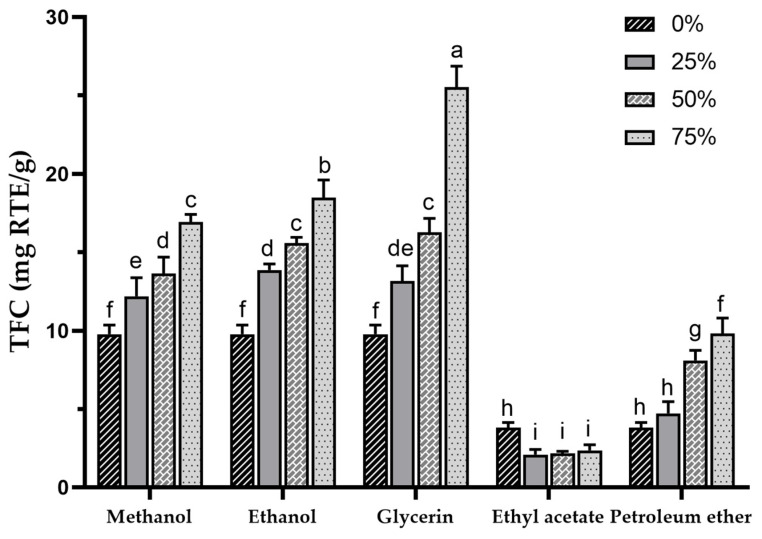
Total flavonoid content of sea buckthorn pomace extracts obtained with different solvents. Different lowercase characters represent significant difference at *p* < 0.05 by Tukey’s multiple range test.

Sea buckthorn contains a high concentration of flavonoids, which are a major class of phenolic compounds. In fact, the TFC in sea buckthorn can be several times higher than that of other plants with high flavonoid contents, which makes SBP a promising resource for development and utilization [1,56]. TFC in various extracts was determined using colorimetry with rutin as a standard. The TFC in different extracts is presented in Figure 2, with the highest TFC found in the 75% glycerol extract (25.52 ± 1.35 mg RTE/g) and the lowest in the 25% ethyl acetate extract (2.13 ± 0.28 mg RTE/g), showing highly significant differences among the various extract TFCs (*p* < 0.001). In a previous study, mechanochemical-assisted extraction with response surface optimization was used to increase the TPC of SBP to 26.82 ± 0.53 mg/g [33], which was similar to the TFC of the 75% glycerol extract in the present study. In this study, the glycerol extracts exhibited the highest TFC (based on the mean values), followed by methanol, ethanol, water, petroleum ether, and ethyl acetate. 

The TFC results are similar to the TPCs, but it is noteworthy that the TFC of the glycerol extracts was higher than that of the methanol and ethanol extracts, indicating a better potential for flavonoid extraction with a concentration-dependent increase. Additionally, the methanol and ethanol groups also displayed higher TFCs, showing a trend similar to TPCs. The use of 50% ethanol in the ultrasound-assisted extraction of SBP was reported to result in a TFC of 11.22 ± 1.89 mg RTE/g [38]. It is possible to achieve a higher TFC by using higher concentrations of ethanol. Compared to the TPC of 75% ethanol extract (18.5 ± 1.12 mg RTE/g), 100% ethanol extract (i.e., 0% ethyl acetate extract, 3.81 ± 0.32 mg RTE/g) showed a significant decrease in TFC, suggesting that pure organic solvents may not be effective for flavonoid extraction, which is consistent with a previous report [57], and this may also be the reason for the lower TFC in petroleum ether and ethyl acetate extracts. For the petroleum ether group, contrary to the TPC results, an increase in TFC was observed with higher petroleum ether concentrations, and the TFC in the 75% petroleum ether extract exceeded that of the water extract, indicating that certain flavonoids in SBP may possess lipophilicity.

Studies have demonstrated that sea buckthorn flavonoids possess physiological benefits, including the improvement of hyperlipidaemia, neuroprotection, hepatoprotection, and the reduction of skin inflammation [33,58,59,60]. The synthesis of flavonoids in sea buckthorn is primarily regulated by 75 genes [61]. Additionally, dehydration stress induced by the drying process increases the synthesis of sea buckthorn flavonoids [62], which may result in an increase in TFC in the dried SBP. Currently, the main solvents used for sea buckthorn flavonoid extraction are aqueous ethanol, while petroleum ether is used for the decolorization and degreasing of extracts [63,64,65]. However, according to our experimental results, petroleum ether extraction may lead to the loss of certain flavonoids. Glycerol, a non-toxic green solvent, has been reported to have a high extraction efficiency in the extraction of plant phenolics [66,67,68]. Furthermore, glycerol-based deep eutectic solvents (DES) have been extensively researched for the extraction of plant phenolics [69,70]. However, the high viscosity and low volatility of glycerol may limit its application.

### 3.3. Antioxidant Activity

#### 3.3.1. DPPH Radical Scavenging Assay

Different solvents not only affect the extraction yield of bioactive substances but also impact the functionality of these compounds. DPPH, a compound containing a nitrogen radical, is easily destroyed by free radical scavengers and is widely used to test the ability of antioxidants to act as proton radical scavengers or hydrogen donors [71]. The antioxidant activity of SBP extracts was tested by DPPH assay. The results, expressed in Torlox equivalents (mg TE/g), are shown in Table 1.

At 75% concentration, ethanol extract exhibited the highest DPPH radical scavenging activity of 13.93 ± 0.41 mg TE/g, followed by methanol and glycerol extracts at 9.92 ± 0.24 mg TE/g and 7.39 ± 0.12 mg TE/g. Previous reports on the ultrasound-assisted extraction of sea buckthorn berries have shown higher DPPH assay results (16.72 ± 0.70 mg TE/g) [72]. However, our results are higher than those reported for the consecutive extraction by pressurized ethanol or water of SBP [37]. The 75% ethyl acetate and 75% petroleum ether extracts exhibited lower scavenging activity, both below the water extract (2.26 ± 0.39 mg TE/g), at 0.63 ± 0.19 mg TE/g and 0.14 ± 0.03 mg TE/g. The type and concentration of solvent exhibited highly significant effects (*p* < 0.001) on the DPPH scavenging activity of SBP extracts. Similar to the TPC results, the ethanol, methanol, and glycerol extracts showed an increase in DPPH radical scavenging activity that was dependent on their concentration. In contrast, the ethyl acetate and petroleum ether extracts showed a decrease in DPPH radical scavenging activity with increasing concentration.

#### 3.3.2. ABTS Radical Scavenging Assay

The ABTS radical scavenging assay is a more effective method for evaluating the antioxidant capacity of hydrophilic, lipophilic, and highly pigmented antioxidants compared to the DPPH assay [73]. To better assess the antioxidant activity of the SBP extracts, the scavenging capacity of ABTS radicals was tested. The results, expressed in Trolox equivalents (mg TE/g), showed significant variations (*p* < 0.001) in the ABTS radical scavenging capacities of extracts with different types and concentrations of solvents, ranging from 0.55 ± 0.66 mg TE/g (75% petroleum ether) to 84.22 ± 1.31 mg TE/g (75% ethanol). Overall, the differences observed in the ABTS assay were greater than those in the DPPH assay, possibly due to the ability of ABTS radicals to evaluate the antioxidant activity of both hydrophilic and lipophilic compounds. In the ABTS assay, methanol and ethanol extracts continued to exhibit strong radical scavenging abilities. The ABTS radical scavenging activity of the 75% ethanol extract (84.22 ± 1.31 mg TE/g) was three times higher than that of the 75% glycerol extract (27.44 ± 1.35 mg TE/g). This difference was greater than that observed in the DPPH assay, indicating that the ABTS assay may not be a reliable method for evaluating antioxidants in glycerol extracts of SBP. In comparison to the results of the ABTS assay (3.58 ± 0.36~1.12 ± 0.40 mmol TE/100 g DM) reported by Tkacz et al. for six different sea buckthorn varieties [3], the SBP extracts in this study exhibited a higher capacity for scavenging free radicals.

#### 3.3.3. Ferric-Reducing Antioxidant Power (FRAP)

The reducing power of a compound can be used to determine its antioxidant capacity. A higher reducing power indicates a greater ability to provide electrons, which makes it easier for free radicals to accept donor electrons and form stable substances, thereby terminating free radical chain reactions [73]. This study employed the FRAP essay, where antioxidants in the extracts reduced the Fe + 3/TPTZ complex to its ferrous form, yielding results compared to ferrous sulfate as a reference standard. This study found that the most effective extract in terms of reducing power was 75% ethanol extract (96.81 ± 1.33 mg FE/g), while the least effective was 75% petroleum ether extract (1.58 ± 0.56 mg FE/g). The results of the FRAP experiment exhibited a comparable trend to that of the DPPH and ABTS essays. The extracts of ethanol, methanol, and glycerol showed a higher reducing power and increased in a concentration-dependent manner.

Based on the antioxidant activity experiments, the 75% ethanol extract of SBP exhibited the highest free radical scavenging and reducing abilities. Overall, ethanol extracts demonstrated the strongest antioxidant activity, followed by methanol extracts with relatively strong antioxidant activity, and glycerol extracts with moderate antioxidant activity, while ethyl acetate and petroleum ether extracts showed poor antioxidant activity. The variation in antioxidant activity among solvent extracts may be attributed to differences in TPC or the composition of their antioxidant components. Water non-soluble fractions may contribute more to the antioxidant activities of SBP [19]. The antioxidant activity of phenolics is determined by their chemical structure, which is influenced by the type of solvent used in the extracts [74]. Solvents of different polarities can dissolve plant chemical substances with similar polarity indices [75], and the polarity of extracts can also impact antioxidant activity [76]. Our findings indicate that SBP is an effective source of natural antioxidants, with ethanol–water extracts exhibiting higher antioxidant activity.

### 3.4. The Phenolic Profiles of Extracts

To investigate the effects of various solvents on the monomeric phenolics in the SBP extracts, we used UPLC-MS/MS to detect and quantify the phenolic profile in different extracts. In total, 13 phenolics were detected in the SBP extracts, as shown in Table 2, including three flavonols (quercetin, isorhamnetin, rutin), one flavanone (naringenin), and one flavanol (epigallocatechin), four cinnamic acids (ferulic acid, coumalic acid, sinapinic acid, chlorogenic acid) and four benzoic acids (gallic acid, protocatechuic acid, vanillic acid, hydroxybenzoic acid).

The content of the 13 phenolic compounds in SBP extracts ranged from high to low as follows: rutin (4.7 ± 2.03~192.21 ± 8.19 μg/g), epigallocatechin (3.8 ± 0.45~105.49 ± 0.69 μg/g), protocatechuic acid (6.79 ± 1.56~27.9 ± 2.38 μg/g), isorhamnetin (2.19 ± 0.33~20.01 ± 0.63 μg/g), coumalic acid (5.62 ± 0.14~10.37 ± 0.23 μg/g), sinapinic acid (5.58 ± 0.16~8.14 ± 0.1 μg/g), hydroxybenzoic acid (5.09 ± 0.13~8.45 ± 0.34 μg/g), gallic acid (2.13 ± 0.1~11.25 ± 0.06 μg/g), vanillic acid (0.00~5.91 ± 0.67 μg/g), quercetin (0.00~5.33 ± 0.07 μg/g), ferulic acid (1.89 ± 0.09~ 2.88 ± 0.12 μg/g), naringenin (0.00~2.04 ± 0.16 μg/g), and chlorogenic acid (0.00~1.55 ± 0.05 μg/g). Sea buckthorn berries from the western Himalayas of India were also identified to have a high content of rutin (155.00~52.20 μg/g) [72]. It has also been reported that isorhamnetin, quercetin, and kaempferol are the main phenolic substances in sea buckthorn [37,77,78].

Among the 13 phenolics, naringenin and quercetin were not detected in water extract, while chlorogenic acid was absent in pure organic solvent extracts, suggesting that neither water nor pure organic solvents are ideal for extracting bioactive substances from SBP. The levels of gallic acid, rutin, isorhamnetin and epigallocatechin were more variable in SBP extracts with different solvents. This suggests that these four substances are more susceptible to the effects of solvents, while several other phenolics are less affected.

The extracts that contained the highest amount of 13 phenolics were 75% glycerol extract (401.53 ± 11.81 μg/g), 50% ethanol extract (278.3 ± 11.11 μg/g), and 75% methanol extract (244.66 ± 14.85 μg/g), in that order, while 75% petroleum ether extract (56.72 ± 3.25 μg/g) exhibited the lowest total amount. In contrast, pure organic solvents (ethyl acetate and petroleum ether extracts) exhibited lower extraction rates of phenolics, even lower than water extract. Importantly, among the 13 phenolics, their highest content was consistently observed in the 75% glycerol extract, which aligns with the results of TFC. This suggests that the glycerol–water solution has significant potential for extracting the bioactive substances of SBP.

Using 75% glycerol extract as a reference, flavonoids were the most dominant phenolic content (325.08 ± 8.31 μg/g) in SBP, with flavonols being the most abundant (217.55 ± 7.63 μg/g) among the flavonoids. Out of the 13 detected phenolic compounds, eight were phenolic acids, but their total amount was not high (76.46 ± 3.53 μg/g). Previous studies have reported that flavonols are the primary polyphenols in sea buckthorn berries [79]. Twenty-eight phenolic substances have been identified from six varieties of sea buck-thorn, twentry-six of which are flavonol derivatives [41].

### 3.5. Correlation Analysis

To investigate the contribution of TPC, TFC, and individual phenolic compounds to the antioxidant activity of SBP extracts, the correlation between TPC, TFC, 13 phenolic compounds, and antioxidant activity was analyzed, as shown in Table 3. A strong correlation was found between TPC (0.916 < r < 0.951) and TFC (0.575 < r < 0.724) with antioxidant activity, with the TPC showing a higher correlation with antioxidant activity, similar to a previous report [53]. This indicates that phenolic compounds might be the primary antioxidant components in SBP extracts, and other phenolic compounds apart from flavonoids also contribute to the antioxidant activity. Studies on ginger and walnut kernels have also reported positive linear correlations between phenolics and antioxidant activity [80,81].

The impact of individual phenolic compounds on the antioxidant activity of SBP can be indirectly reflected by the correlation between their content and antioxidant capacity. Nine phenolics showed a significant correlation (*p* < 0.05) with antioxidant activities. Among these, hydroxybenzoic acid, vanillic acid, and rutin showed the highest correlation, indicating that phenolic acids and flavonoids collectively jointly contribute to the antioxidant properties of SBP. Rutin, as the p pound with the highest content detected in SBP extracts, also exhibited a significant correlation with antioxidant activity (*p* < 0.05), indicating that rutin might be the primary antioxidant compound in SBP, highlighting its importance in future research focusing on the antioxidant properties of SBP.

Multivariate analyses were conducted using the orthogonal projection to latent structure-discriminant analysis (OPLS-DA) to evaluate the variations in phenolic profiles and antioxidant activities among different SBP extracts. For the OPLS-DA model, R^2^X and R^2^Y are commonly used to assess the model’s goodness of fit and reliability, while Q^2^ is typically used to evaluate its predictive ability [82]. The study’s model parameters, R^2^X, R^2^Y, and Q^2^, had values of 0.751, 0.755, and 0.636, which indicated a good model fit and predictability. The permutation testing of the model indicated a significantly high level of predictive accuracy (*p* < 0.05).

**Figure 3:**
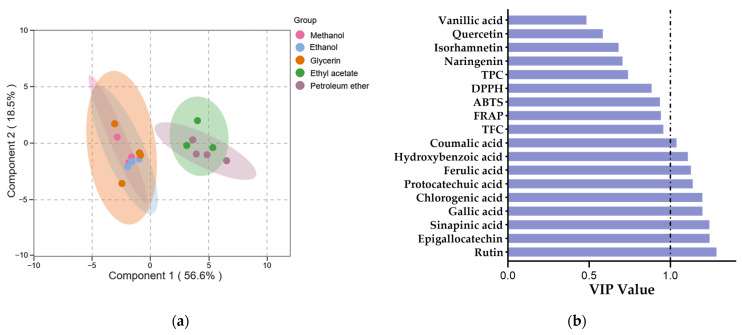
The OPLS-DA of phenolic profile and antioxidant activity of SBP extracts with different solvents. (**a**) Score plot of OPLS-DA. (**b**) VIP plot of OPLS-DA.

The score plot generated by OPLS-DA revealed a clearer segregation was achieved among the methanol, ethanol, glycerol extracts and the ethyl acetate, petroleum ether extracts. (Figure 3a), which indicated significant differences in the phenolic profiles and antioxidant activities of pure organic solvent extracts and aqueous organic solvent extracts. The variable importance in the projection (VIP) is typically used to explain the contribution of variables to the model, variables with VIP > 1 are considered to have the greatest impact on the model [83]. The VIP values for all variables are displayed in Figure 3b. The higher the VIP value, the greater the difference in phenolic content between the extracts. Nine phenolic compounds were identified with VIP values greater than 1, indicating their potential use as markers to differentiate between various solvent SBP extracts.

**Figure 4:**
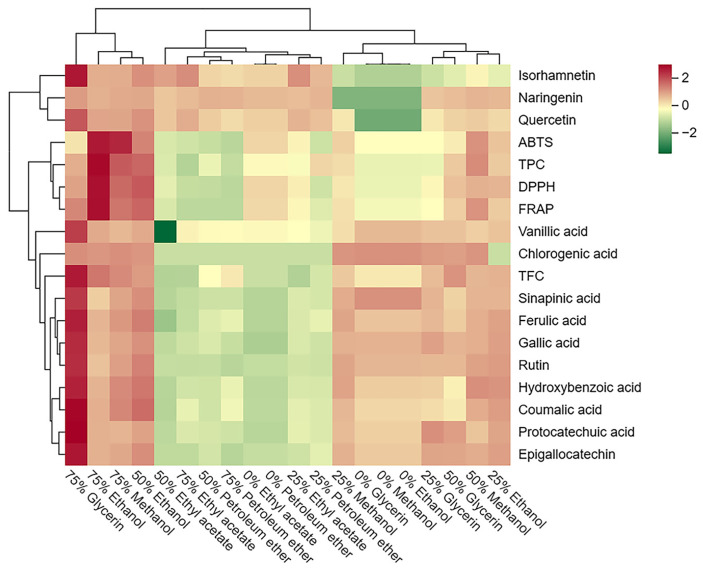
Cluster heatmap of the phenolic profile and antioxidant activity of different solvent extracts from SBP. Mean values refer to colors from minimum displayed in green to maximum represented with red.

Further visualization through a cluster heatmap illustrated the concentration and composition of phenolic compounds, and antioxidant activities in SBP extracts with different solvents (Figure 4). The cluster heatmap demonstrated significant differences between SBP extracts obtained using different solvents. Vertically, the extracts of 75% glycerol, 75% ethanol, 50% ethanol, and 75% methanol were grouped due to their higher content of phenolic compounds and antioxidant activity, and the 75% ethanol extract exhibited the most prominent antioxidant activity. Conversely, all petroleum ether and ethyl acetate extracts were grouped together due to their lower phenolic content and antioxidant activity. Horizontally, three flavonoids (isorhamnetin, naringenin, and quercetin) were separately clustered due to their lower solubility in water. Moreover, TPC was clustered along with the three antioxidant tests, reaffirming the significant contribution of TPC to antioxidant activity in SBP. 

## 4. Conclusions

The utilization of by-products from food processing is a crucial issue and a necessary path for the further development of the food industry. SBP, an excellent source of natural antioxidants, holds significant value for added utilization in industries like food and cosmetics. To provide a basis for the value-added utilization of SBP, this study investigated the active compounds and antioxidant activities of SBP extracts using different solvents. The results indicate that SBP extracts are a rich source of phenolic compounds and natural antioxidants, with a high potential for utilization. Notably, significant differences were observed in the extracts obtained with different solvents. The aqueous extracts of methanol, ethanol, and glycerol with higher concentrations showed higher phenolic content and antioxidant activity compared to pure organic solvents and water. There was a strong correlation between phenolic compounds and antioxidant activities. Flavonols, primarily represented by rutin, were the most abundant phenolic compounds in SBP extracts. Notably, the 75% glycerol aqueous solution reached the highest levels of TFC and the 13 phenolic compounds detected by UPLC-MS/MS among all extraction solutions, demonstrating exceptional extraction efficiency. Glycerol, known for its safety, stability, and eco-friendly characteristics, suggests potential further development for SBP extraction methods targeting glycerol aqueous solutions.

## Figures and Tables

**Table 1:** The antioxidant activities of different solvent extracts of SBP determined by DPPH, ABTS radical scavenging and FRAP assays.

Solvent of Extracts		DPPH (mg TE/g)	ABTS (mg TE/g)	FRAP (mg FE/g)
Methanol	0%	2.26 ± 0.39 ^g^	19.99 ± 1.18 ^i^	18.54 ± 0.58 ^j^
	25%	4.33 ± 0.63 ^ef^	32.83 ± 0.91 ^f^	31.81 ± 1.22 ^g^
	50%	6.76 ± 0.66 ^cd^	50.04 ± 1.63 ^d^	55.88 ± 1.19 ^e^
	75%	9.92 ± 0.24 ^b^	80.25 ± 1.28 ^b^	65.04 ± 1.08 ^c^
Ethanol	0%	2.26 ± 0.39 ^g^	19.99 ± 1.18 ^i^	18.54 ± 0.58 ^j^
	25%	6.53 ± 0.16 ^cd^	36.35 ± 0.89 ^e^	38.32 ± 1.26 ^f^
	50%	10.7 ± 0.72 ^b^	53.96 ± 2.14 ^c^	70.13 ± 1.5 ^b^
	75%	13.93 ± 0.41 ^a^	84.22 ± 1.31 ^a^	96.81 ± 1.33 ^a^
Glycerin	0%	2.26 ± 0.39 ^g^	19.99 ± 1.18 ^i^	18.54 ± 0.58 ^j^
	25%	3.62 ± 0.11 ^f^	20.08 ± 0.32 ^i^	22.51 ± 0.77 ^i^
	50%	6.06 ± 0.14 ^d^	25.41 ± 0.89 ^gh^	37.29 ± 1.43 ^f^
	75%	7.39 ± 0.12 ^c^	27.44 ± 1.35 ^g^	59.64 ± 1.92 ^d^
Ethyl acetate	0%	5.1 ± 0.12 ^e^	31.32 ± 1.10 ^f^	34.31 ± 1.35 ^g^
	25%	4.01 ± 0.13 ^f^	23.43 ± 0.50 ^h^	25.14 ± 1.60 ^h^
	50%	1.95 ± 0.03 ^g^	8.69 ± 0.67 ^j^	10.09 ± 1.29 ^k^
	75%	0.63 ± 0.19 ^hi^	6.66 ± 0.54 ^jk^	1.62 ± 1.44 ^l^
Petroleum ether	0%	5.1 ± 0.12 ^e^	31.32 ± 1.10 ^f^	34.31 ± 1.35 ^g^
	25%	1.04 ± 0.19 ^h^	5.54 ± 0.66 ^kl^	11.92 ± 1.13 ^k^
	50%	0.49 ± 0.06 ^hi^	4.14 ± 0.36 ^l^	1.76 ± 0.53 ^l^
	75%	0.14 ± 0.03 ^i^	0.55 ± 0.66 ^m^	1.58 ± 0.56 ^l^

All values are the mean ± SD (*n* = 3). The superscript in each column represents significant *p* values (*p* < 0.05) that are different from each other.

**Table 2:** Identifications and quantifications of the phenolic components from sea buckthorn pomace extracts with different solvents.

Solvent of Extracts		Gallic Acid	Ferulic Acid	Naringenin	Quercetin	Chlorogenic Acid	Rutin	Protocatechuic Acid
		mg/kg						
Methanol	0%	7.05 ± 0.14 ^de^	2.38 ± 0.08 ^c^	ND	ND	1.52 ± 0.07 ^ab^	100.67 ± 4.64 ^de^	14.47 ± 1.55 ^d^
	25%	7.10 ± 0.09 ^de^	2.47 ± 0.04 ^bc^	ND	2.85 ± 0.15 ^h^	1.48 ± 0.01 ^bc^	112.04 ± 5.18 ^cd^	15.73 ± 1.55 ^bcd^
	50%	7.17 ± 0.08 ^d^	2.44 ± 0.02 ^bc^	1.81 ± 0.16 ^bcde^	3.33 ± 0.12 ^efg^	1.48 ± 0.04 ^bc^	113.77 ± 13.25 ^c^	15.20 ± 2.45 ^cd^
	75%	7.42 ± 0.10 ^c^	2.51 ± 0.10 ^bc^	1.90 ± 0.02 ^bc^	3.99 ± 0.15 ^c^	1.53 ± 0.10 ^ab^	115.87 ± 12.07 ^c^	16.20 ± 1.00 ^bcd^
Ethanol	0%	7.05 ± 0.14 ^de^	2.38 ± 0.08 ^c^	ND	ND	1.52 ± 0.07 ^ab^	100.67 ± 4.64 ^de^	14.47 ± 1.55 ^d^
	25%	7.63 ± 0.09 ^c^	2.48 ± 0.09 ^bc^	1.78 ± 0.03 ^bcdef^	3.10 ± 0.16 ^fgh^	ND	118.25 ± 11.66 ^c^	17.17 ± 2.13 ^bcd^
	50%	8.08 ± 0.06 ^b^	2.59 ± 0.07 ^b^	1.92 ± 0.10 ^ab^	4.32 ± 0.21 ^b^	1.47 ± 0.04 ^bc^	135.90 ± 10.08 ^b^	17.41 ± 1.99 ^bc^
	75%	6.88 ± 0.10 ^e^	2.42 ± 0.08 ^c^	1.84 ± 0.05 ^bcd^	4.00 ± 0.13 ^c^	1.47 ± 0.05 ^bc^	93.22 ± 8.76 ^e^	16.41 ± 0.94 ^bcd^
Glycerin	0%	7.05 ± 0.14 ^de^	2.38 ± 0.08 ^c^	ND	ND	1.52 ± 0.07 ^ab^	100.67 ± 4.64 ^de^	14.47 ± 1.55 ^d^
	25%	7.61 ± 0.21 ^c^	2.41 ± 0.08 ^c^	1.67 ± 0.02 ^ef^	2.83 ± 0.10 ^h^	1.43 ± 0.03 ^cd^	98.59 ± 8.63 ^de^	18.56 ± 0.76 ^b^
	50%	6.96 ± 0.15 ^de^	2.35 ± 0.04 ^c^	1.73 ± 0.06 ^def^	3.21 ± 0.04 ^efg^	1.39 ± 0.04 ^d^	99.25 ± 8.54 ^de^	17.67 ± 1.68 ^bc^
	75%	11.25 ± 0.06 ^a^	2.88 ± 0.12 ^a^	2.04 ± 0.16 ^a^	5.33 ± 0.07 ^a^	1.55 ± 0.05 ^a^	192.21 ± 8.19 ^a^	27.90 ± 2.38 ^a^
Ethyl acetate	0%	2.13 ± 0.10 ^j^	1.97 ± 0.15 ^de^	1.76 ± 0.08 ^cdef^	3.26 ± 0.07 ^efg^	ND	11.61 ± 2.73 ^fg^	6.79 ± 1.56 ^e^
	25%	3.49 ± 0.09 ^f^	2.08 ± 0.10 ^d^	1.73 ± 0.14 ^def^	3.73 ± 0.06 ^cd^	ND	20.64 ± 3.00 ^f^	9.57 ± 0.68 ^e^
	50%	2.71 ± 0.11 ^i^	1.89 ± 0.09 ^e^	1.66 ± 0.06 ^f^	3.41 ± 0.08 ^ef^	ND	13.15 ± 2.23 ^fg^	7.08 ± 0.60 ^e^
	75%	3.14 ± 0.06 ^g^	2.01 ± 0.19 ^de^	1.75 ± 0.03 ^cdef^	3.85 ± 0.16 ^c^	ND	12.01 ± 1.28 ^fg^	8.93 ± 1.13 ^e^
Petroleum ether	0%	2.13 ± 0.10 ^j^	1.97 ± 0.15 ^de^	1.76 ± 0.08 ^cdef^	3.26 ± 0.07 ^efg^	ND	11.61 ± 2.73 ^fg^	6.79 ± 1.56 ^e^
	25%	3.18 ± 0.06 ^g^	2.12 ± 0.07 ^d^	1.81 ± 0.04 ^bcdef^	3.52 ± 0.46 ^de^	ND	18.27 ± 3.44 ^fg^	9.00 ± 1.56 ^e^
	50%	3.51 ± 0.11 ^f^	2.09 ± 0.06 ^d^	1.84 ± 0.02 ^bcd^	3.31 ± 0.22 ^efg^	ND	12.94 ± 4.11 ^fg^	8.60 ± 0.69 ^e^
	75%	2.94 ± 0.25 ^h^	2.12 ± 0.03 ^d^	1.83 ± 0.07 ^bcd^	3.05 ± 0.10 ^gh^	ND	4.70 ± 2.03 ^g^	8.19 ± 0.83 ^e^
**Solvent of Extracts**		**Vanillic Acid**	**Hydroxybenzoic Acid**	**Coumalic Acid**	**Sinapinic Acid**	**Isorhamnetin**	**Epigallocatechin**	**Total Amount**
		**mg/kg**						
Methanol	0%	4.29 ± 0.09 ^b^	6.49 ± 0.24 ^e^	7.29 ± 0.20 ^e^	7.31 ± 0.27 ^b^	2.19 ± 0.33 ^j^	45.81 ± 0.49 ^f^	199.46 ± 3.06 ^f^
	25%	3.80 ± 0.40 ^bc^	6.95 ± 0.15 ^cd^	7.75 ± 0.26 ^d^	7.06 ± 0.12 ^c^	3.58 ± 0.26 ^i^	53.74 ± 0.46 ^e^	224.56 ± 3.10 ^de^
	50%	4.01 ± 0.30 ^bc^	7.19 ± 0.16 ^bc^	7.92 ± 0.20 ^d^	6.97 ± 0.08 ^c^	7.37 ± 0.17 ^g^	54.92 ± 0.94 ^e^	233.57 ± 13.43 ^cd^
	75%	4.3 ± 0.55 ^b^	7.25 ± 0.03 ^bc^	8.46 ± 0.36 ^bc^	7.12 ± 0.13 ^bc^	11.43 ± 0.56 ^d^	56.68 ± 0.79 ^d^	244.66 ± 14.85 ^c^
Ethanol	0%	4.29 ± 0.09 ^b^	6.49 ± 0.24 ^e^	7.29 ± 0.20 ^e^	7.31 ± 0.27 ^b^	2.19 ± 0.33 ^j^	45.81 ± 0.49 ^f^	199.46 ± 3.06 ^f^
	25%	4.15 ± 0.41 ^bc^	7.14 ± 0.12 ^c^	8.14 ± 0.14 ^cd^	6.98 ± 0.16 ^c^	5.20 ± 0.29 ^h^	59.55 ± 1.60 ^c^	241.57 ± 12.16 ^c^
	50%	4.49 ± 0.40 ^b^	7.48 ± 0.20 ^b^	8.86 ± 0.17 ^b^	7.34 ± 0.19 ^b^	13.08 ± 0.36 ^b^	65.37 ± 1.69 ^b^	278.30 ± 11.11 ^b^
	75%	4.49 ± 0.11 ^b^	6.77 ± 0.06 ^de^	7.87 ± 0.29 ^d^	6.74 ± 0.11 ^d^	11.36 ± 0.08 ^d^	53.70 ± 2.32 ^e^	217.17 ± 12.06 ^e^
Glycerin	0%	4.29 ± 0.09 ^b^	6.49 ± 0.24 ^e^	7.29 ± 0.20 ^e^	7.31 ± 0.27 ^b^	2.19 ± 0.33 ^j^	45.81 ± 0.49 ^f^	199.46 ± 3.06 ^f^
	25%	4.18 ± 0.94 ^bc^	6.45 ± 0.44 ^e^	7.20 ± 0.45 ^e^	6.98 ± 0.16 ^c^	3.77 ± 0.29 ^i^	57.64 ± 0.46 ^d^	219.34 ± 7.10 ^de^
	50%	4.18 ± 0.36 ^bc^	6.08 ± 0.13 ^f^	7.02 ± 0.24 ^e^	6.70 ± 0.13 ^d^	4.99 ± 0.16 ^h^	57.35 ± 0.95 ^d^	218.88 ± 8.25 ^de^
	75%	5.91 ± 0.67 ^a^	8.45 ± 0.34 ^a^	10.37 ± 0.23 ^a^	8.14 ± 0.10 ^a^	20.01 ± 0.63 ^a^	105.49 ± 0.69 ^a^	401.53 ± 11.81 ^a^
Ethyl acetate	0%	3.46 ± 0.35 ^bc^	5.12 ± 0.12 ^hi^	5.71 ± 0.21 ^h^	5.58 ± 0.16 ^f^	9.32 ± 0.37 ^f^	8.68 ± 0.44 ^i^	65.40 ± 2.33 ^ij^
	25%	3.36 ± 0.62 ^bc^	5.45 ± 0.17 ^gh^	6.27 ± 0.23 ^fg^	5.93 ± 0.09 ^e^	13.13 ± 0.32 ^b^	11.18 ± 0.40 ^h^	86.56 ± 3.29 ^gh^
	50%	ND	5.09 ± 0.13 ^i^	5.62 ± 0.14 ^h^	5.59 ± 0.02 ^f^	12.10 ± 0.32 ^c^	6.54 ± 0.28 ^j^	64.83 ± 2.66 ^ij^
	75%	3.55 ± 0.45 ^bc^	5.38 ± 0.05 ^ghi^	6.34 ± 0.21 ^fg^	5.70 ± 0.18 ^ef^	13.25 ± 0.27 ^b^	6.54 ± 0.14 ^j^	72.44 ± 1.51 ^hi^
Petroleum ether	0%	3.46 ± 0.35 ^bc^	5.12 ± 0.12 ^hi^	5.71 ± 0.21 ^h^	5.58 ± 0.16 ^f^	9.32 ± 0.37 ^f^	8.68 ± 0.44 ^i^	65.40 ± 2.33 ^ij^
	25%	3.12 ± 1.09 ^c^	5.44 ± 0.05 ^gh^	6.16 ± 0.08 ^fg^	5.87 ± 0.06 ^e^	10.77 ± 0.39 ^e^	18.43 ± 0.05 ^g^	87.68 ± 4.68 ^g^
	50%	3.43 ± 0.90 ^bc^	5.35 ± 0.05 ^ghi^	5.99 ± 0.09 ^gh^	5.80 ± 0.03 ^ef^	9.21 ± 0.40 ^f^	12.44 ± 0.28 ^h^	74.51 ± 5.18 ^ghi^
	75%	3.41 ± 0.89 ^bc^	5.65 ± 0.16 ^g^	6.46 ± 0.29 ^f^	5.80 ± 0.06 ^ef^	8.77 ± 0.07 ^f^	3.80 ± 0.45 ^k^	56.72 ± 3.25 ^j^

All values are the mean ± SD (*n* = 3). ND—not detectable. The superscript in each column represents significant *p* values (*p* < 0.05) that are different from each other.

**Table 3:** Pearson correlation analysis of TPC, TFC and individual phenolic compounds with antioxidant activity.

	DPPH	ABTS	FRAP
**TPC**	0.937 **	0.916 **	0.951 **
**TFC**	0.688 **	0.575 *	0.724 **
**Gallic acid**	0.618 **	0.517 *	0.653 **
**Ferulic acid**	0.635 **	0.542 *	0.680 **
**Naringenin**	0.227	0.110	0.200
**Quercetin**	0.405	0.263	0.406
**Chlorogenic acid**	0.088	0.208	0.199
**Rutin**	0.658 **	0.564 *	0.694 **
**Protocatechuic acid**	0.588 *	0.446	0.625 **
**Vanillic acid**	0.602 *	0.440	0.643 **
**Hydroxybenzoic acid**	0.667 **	0.597 *	0.714 **
**Coumalic acid**	0.669 **	0.572 *	0.713 **
**Sinapinic acid**	0.583 *	0.506 *	0.627 **
**Isorhamnetin**	0.211	0.084	0.239
**Epigallocatechin**	0.653 **	0.531 *	0.691 **

Significance level: *p* < 0.05 (*), *p* < 0.01 (**).

## Data Availability

The original contributions presented in the study are included in the article, further inquiries can be directed to the corresponding author.
